# Behavioral differences of individuals with different self-regulation levels in a real-life example of teamwork—DOTA 2

**DOI:** 10.3389/fpsyg.2022.1054675

**Published:** 2022-11-28

**Authors:** Yilin Wang, Jiexing Leng, Yichuan Zhang, Wenwen Chen, Fugui Xing, Nan Zhao

**Affiliations:** ^1^CAS Key Laboratory of Behavioral Science, Institute of Psychology, Chinese Academy of Sciences, Beijing, China; ^2^Department of Psychology, University of Chinese Academy of Sciences, Beijing, China; ^3^National Time Service Center, Chinese Academy of Sciences, Xi'an, China; ^4^School of Integrated Circuits, University of Chinese Academy of Sciences, Beijing, China; ^5^Faculty of Psychology, Beijing Normal University, Beijing, China

**Keywords:** teamwork, self-regulation, gaming behavior, DOTA 2, MOBA

## Abstract

Teamwork is a vital aspect of human life, including a set of concrete behaviors which could be divided into various categories such as task performance, job dedication, backing up behavior, and monitoring. As an essential psychological factor could form team members to adapt to environmental changes, self-regulation has a marked impact on teamwork results. However, why self-regulation could affect results of teamwork in real life and how self-regulation influence the concrete teamwork behaviors remains unclear. This study recorded and extracted participants’ detailed gaming behaviors in Defense of the Ancients 2 (DOTA 2), which is an example of real-life teamwork scenario. The sample consisted of 59 DOTA 2 players with relative low-level self-regulation (93.22% male) and 59 with relative high-level self-regulation (96.61% male). Controlling confounding factors, we explored behavioral differences between the two groups in different types of heroes. Results showed that self-regulation influenced specific gaming behaviors including the categories of task performance, job dedication, and backing up behavior, but not including monitoring. Additionally, these impacts of self-regulation varied by hero type. These results demonstrate the different impacts of self-regulation on different categories of teamwork behaviors, and these impacts are considerably determined by individual’s role in the team. These findings shed light on the mechanism of the teamwork performance improvement caused by self-regulation and provide new insights into understanding the different impact patterns of self-regulation in different real-life tasks and responsibilities.

## Introduction

Humans have a natural tendency to form various groups with common identities to achieve common results, making teams an essential aspect of human society (Liu et al., 2021). Teams enable individuals to better adapt to complex and changing conditions to achieve a shared goal (Dyer, 1984; Salas et al., 1992). In terms of behavioral performance, teamwork involves multiple behavioral processes that connect variables as team and member with performance quality and other outcome variables (Marks et al., 2001). Borman and Motowidlo (1993) came up with a two-dimensional teamwork performance model, including task performance and contextual performance. Task performance is defined as the work activities that are directly conducted according to specified responsibilities, reflecting the effectiveness with which individuals directly achieve the team’s goal; other than job-specific acts, contextual performance is a set of interpersonal and volitional behaviors that support the social and motivational context in which organizational work is accomplished (Rong et al., 2015). Van Scotter and Motowidlo (1996) further refined the construct of contextual performance by dividing it into two narrower constructs—job dedication and interpersonal facilitation. Job dedication includes self-disciplined, motivated acts such as working hard; interpersonal facilitation refers to the cooperative, considerate and helpful acts that assist co-workers’ performance, which means the backing up behavior (McIntyre and Salas, 1995; Porter et al., 2003) In addition, the changing nature of the teamwork environment requires team members to keep a constant and timely track of contextual changes, making monitoring an essential part of teamwork (Ilgen and Pulakos, 1999; Wen, 2005). Many pieces of research have proved that for teamwork, monitoring is as important as backing up behavior (McIntyre and Salas, 1995; Marks et al., 2001; Salas et al., 2005). Based on these previous studies, at least four different categories of teamwork behaviors could be identified: task performance, job dedication, backing up behavior, and monitoring.

Exploring how psychological feature plays its role in teamwork performance is of vital importance for understanding teamwork behaviors and improving teamwork performance. Self-regulation is defined as the capacity that for altering individual’s behaviors to adapt the environment towards his/her goal (Winne, 2001; Baumeister and Vohs, 2004). As shown in several studies, self-regulation could play an indispensable role in teamwork (Pintrich and Groot, 1990; Zimmerman and Martinez-Pons, 1992; Li and Sun, 2011; Wei et al., 2015). In an experiment by Sachdeva et al. (2009), individuals are inclined to take more cooperative acts when self-regulating themselves to pursue ideal traits. The research by Xu and Luo (2011) also revealed that individuals would harness the self-regulation mechanism in collaborative learning. To effectively improve teamwork performance, self-regulation is needed throughout the process of teamwork, including task preparation, execution and evaluation (Tschan, 1995, 2002; Gevers et al., 2009). However, these researches did not reveal how self-regulation can impact concrete teamwork performance in real-life circumstances of teamwork.

To answer this question, we need to explore team member’s specific behaviors in a real-life teamwork case. Previous researches usually took two methods to study teamwork. One is to set up a laboratory-based teamwork scenario with designed tasks instead of real-life ones, in which individuals are brought together to complete the tasks in lab settings (Tuckman, 1965; Liu et al., 2021). The gap between the invented tasks and the real-life ones means that the results of the laboratory-based method may not produce good ecological validity (Buchanan and Smith, 1999; Carlbring et al., 2007). The other is the self-reporting method, by which participants are interviewed or asked to fill in questionnaires based on recollections of their real-life experiences (Wen, 2005). Though the self-reporting method allows the participants to answer questions based on real-life experiences, it cannot avoid recall bias, leading to the inaccuracy of the recollections retrieved (Bishop et al., 2000), and cannot demonstrate in detail the behavioral differences caused by psychological factors. Due to the limitations of such methods, previous researches focused more on how self-regulation impacted the final results of teamwork, rather than on what roles self-regulation played in various behavior processes in real-life teamwork (Sachdeva et al., 2009; Xu and Luo, 2011).

Defense of the Ancients 2 (DOTA 2) is a multiplayer online battle arena (MOBA) game and enjoys high popularity globally (Steamcharts, 2021). It is played in matches between two teams of five players, with each team member controlling a “hero.” To defend their own base and destroy the opponent’s base, players of each team need to cooperate with each other, which provides a typical real-life teamwork scenario. We can observe many specific teamwork behaviors of various categories in DOTA 2 matches, for example: “kill” means killing opposing heroes, a typical behavior of task performance; “buying items” means players purchase new equipment to enhance the ability of their heroes, a typical behavior of job dedication; “team fight” refers to the situation in which two teams compete with each other, with each team having at least two players to support each other, a typical behavior of backing up behavior; “placing wards” means players place wards to gain better vision and monitor a certain area, a typical behavior of monitoring. During the game, all these behaviors of players are recorded in a real-time and accurate way, providing us with detailed and complete data for studying real-life teamwork behaviors.

Considering these advantages of DOTA 2, this paper attempted to utilize this game to explore individual behaviors in real-life teamwork settings. We recruited players with different self-regulation levels, extracted their behavior data recorded in a real-time manner, and studied how self-regulation could impact teamwork behaviors. More specifically, since teamwork depends on self-regulation to achieve goals, and it contains several behavioral categories (Priest et al., 2002), this study examined whether and to what extent players with different self-regulation levels differ in terms of their: (1) task performance, (2) job dedication, (3) backing up behavior, and (4) monitoring. We hypothesized that self-regulation could impact all these behaviors of different categories in real-life teamwork settings, DOTA 2, and the impact was different between each category.

## Materials and methods

### Research tool

#### Short version of the self-regulation questionnaire

We utilized the short version of the self-regulation questionnaire (SSRQ; α = 0.92) developed by Carey et al. (2004). Each item is scored by 5-point Likert scale, and the answers range from 1 (strongly disagree) to 5 (strongly agree). There are 31 items in total, of which 17 items are scored positively and 14 items are scored inversely.

#### DOTA 2

This study used version 7.29 of DOTA 2, which is played in matches between two teams of five players. In the game fight environment, the opponents and the teammates have an impact on the gaming processing and behaviors. This study controlled variables in map, the strength of opponents and teammates in DOTA 2 by unifying settings of the game room (Lyu et al., 2021).

### Participants and data collection

We recruited participants from online game BBS and groups after obtaining their informed consent. The experimental process is as follows:

1. Participants filled out and submitted the online questionnaire, which included basic information related to DOTA 2 (e.g., “What is your rank in Dota 2?”; “What is your steam ID?”), demographic information (e.g., age and sex), and SSRQ.
2. Participants logged into version 7.29 of DOTA 2 to play a game. Through screen sharing, these participants were supervised by the experimenter to change default lobby settings as follows: clicking “filling empty slots with bots,” “hard bot difficulty,” and “all pick.”
3. After the game, participants saved and submitted the log file under the guidance of the experimenter.
4. The experimenter downloaded the total time of their all competitions synchronized on DOTA 2 by logging into the steam account provided by the participants.

Privacy was strictly protected during this process, reference to the ethical principles listed by Kosinski et al. (2015). This study was approved by the scientific research ethics committee of Institute of Psychology, Chinese Academy of Sciences (ethical code H15010).

We selected players who met the following criteria as our research participants:

1. Spent more than 300 s on completing the questionnaire and passed screening questions (e.g., “Please choose 2020 for this question”).
2. Played more than 25 min in this round of the game to ensure that they take the game seriously.

Finally, we obtained 218 effective participants aged between 18 and 32 years old, with an average age of 22.98 and a standard deviation of 2.23. Among them, 209 were male (age = 23.00 ± 2.25) and 9 were female (age = 22.44 ± 1.67).

### Gaming behavior measurement

#### Gaming behavior data

In this study, we used the program provided by DOTA 2 official website^1^ to convert the collected game video files into data tables. We calculated 18 indicators of player’s specific behaviors during this game from the data tables. The details of the calculation can be seen in Lyu et al. (2021). Based on the criteria of the four categories mentioned in Introduction, these 18 indicators could be classified into the four categories as follows:

1. The behavior indicators belonging to the category of task performance all cause a direct damage to the enemy and clearly reflect whether their own team has advantages in the fight.
2. The behavior indicators belonging to the category of job dedication do not have a direct impact on the enemy, but they all reflect the player’s motivation to improve his or her ability to deal with the enemy’s attack. These behavior indicators reflect the motivation basis of task performance, and drive players to promote the realization of team goals.
3. The behavior indicators belonging to the category of backing up behavior all reflect the player’s interpersonal-oriented behaviors to cooperate with and help others to complete tasks.
4. The behavior indicators belonging to the category of monitoring all reflect the player’s attention to the environment changes, and adjusts his or her behaviors in time.

Table 1 shows specific behavior indicators.

**Table 1:** Gaming behavior indicators.

**Behavior indicator**	**Category**
Proportion of the number of skills used for enemy	Task performance
Number of destroyed defensive towers of enemy	Task performance
Number of kills	Task performance
Average number of kills	Task performance
Number of multi-kills	Task performance
Proportion of the number of skills used for oneself	Job dedication
Number of times to use gold to respawn	Job dedication
Number of purchased comprehensive items	Job dedication
Number of purchased active items	Job dedication
Number of consumables	Job dedication
Total number of team fights on the initial stage	Backing up behavior
Total number of team fights on the middle stage	Backing up behavior
Total number of team fights on the later stage	Backing up behavior
Proportion of the number of skills used for teammates	Backing up behavior
Total number of placed observer wards	Monitoring
Average number of placed observer wards	Monitoring
Total number of placed sentry wards	Monitoring
Average number of sentry wards	Monitoring

#### Confounding factors

Besides self-regulation, there are also some confounding factors that can affect the behaviors in game operation (such as hero type). We needed to measure these factors and control them in further analysis, in order to better clarify the characteristics and patterns of gaming behaviors of individuals with different self-regulation levels (see Table 2).

**Table 2:** Confounding factors.

**Confounding factor**	**Common gaming behavior**
**Hero type** ^**a**^	
Strength	Strength-type heroes are mainly responsible for dealing with the physical damage from the opposing team and controlling the opposing heroes. Moving slowly and heavily without much flexibility, most strength-type heroes are responsible for melee. In the game, strength-type heroes frequently use skills to increase the attack speed, movement speed, health points (HP), physical defense points, magical defense points of their own or their teammates, decrease the HP of opposing heroes, impair their attack speed, movement speed, physical defense points, and magical defense points, and even disable them. When combating together with teammates, strength-type heroes usually stand in the front of the team and deal with heavy physical damage from the opposing team.
Agility	Agility-type heroes tend to deliver the highest physical damage. Moving rapidly with great flexibility, agility-type heroes usually make attacks at middle and remote range. As the team core in the game, agility-type heroes often kill more opposing heroes than their teammates. Agility-type heroes frequently participate in the team fights on the middle and later stage.
Intelligence	Intelligence-type heroes often act as supporting roles for their teammates to deliver physical damage when facing with opposing heroes and typically combat at remote range. In a game, intelligence-type heroes frequently cast spells. They can increase the attack speed, movement speed, HP, physical defense points, magical defense points of their own or their teammates, decrease opposing heroes’ HP.
**Lane** ^**a**^	
Dead	Against the safe lane of the opposing team, the dead lane is where your own creeps get closer to the opposing unit’s defensive towers, thereby making it easier for opposing heroes to kill your hero and making it harder for your hero to kill more lane creeps, neutral creeps and heroes of the opposing team. As a result, fewer experience and gold are gained, and your hero level could be relatively low.
Mid	Being the shortest lane, the mid lane is where you can meet more opposing creeps. Additionally, the distance between the mid lane and the neutral creeps is close, so your hero can kill more creeps and neutral creeps here, gaining more gold and higher level. Mid-lane heroes not only kill opposing heroes along this lane but also support teammates in other lanes to kill opposing heroes.
Safe	Being the longest lane, the safe lane is where the opposing creeps get closer to your defensive towers, making it easier for your heroes to kill more creeps, neutral creeps and opposing heroes. Safe lane heroes can scale well without turning to other lanes.
**Historical game duration** ^**b**^	The longer a player’s historical game duration is, the more skilled the player is at operating various heroes. With more time spent on the game, a player knows better about the responsibilities of his or her hero and acquires more gaming experience.
**Rank**^**c**^	
None	The higher a player’s rank is, the better the player knows about the abilities of each hero. Higher rankers can more flexibly and skillfully control their heroes.
Herald	
Guardian	
Crusader	
Archon	
Legend	
Ancient	
Divine	
Immortal	

a Video recording.

b Game history data.

c Online questionnaire.

As for the confounding factors of hero type and lane, we obtained and analyzed them in the same way as we extracted the gaming behavior indicators shown above. As for the historical game duration, we obtained it by writing a web crawler in Python and downloading the competition history data synchronized on DOTA 2 platform according to the steam account provided by the participants. As for the rank of player, we obtained it through the online questionnaire.

### Data analysis

In order to test the most indubitable difference in gaming behaviors brought by self-regulation level, we firstly sorted the self-regulation scores of 218 participants in a descending order, and selected the top 27% samples as the high-level group and the bottom 27% samples as the low-level group according to the recognized classification standard (Kelley, 1939). The following analysis was conducted only based on high- and low-level group participants. In order to test whether these four confounding factors are independent from the self-regulation level, we firstly tested the historical game duration by an independent sample t-test, and tested hero type, lane and rank in DOTA 2 through crosstabs analysis. Then, we adopted multi-factor analysis of variance (ANOVA) to clarify the differences in gaming behaviors of individuals with different self-regulation levels after controlling the confounding factors.

All statistical tests were performed using SPSS version 26 for Windows.

## Results

### Demographics

Among the 218 valid participants, 59 were screened out and categorized into the low-level group (self-regulation = 58.25 ± 7.812) and another 59 into the high-level group (self-regulation = 98.86 ± 8.423). The selected 118 subjects ranged in age between 18 and 32 years, with a median age of 23.10 (SD = 2.38). Table 3 features the demographic profiles of these participants.

**Table 3:** Demographic characteristics of participants with high- and low-level of self-regulation, n (%).

**Characteristic**	**Self-regulation level**	
	Low (n = 59)	High (n = 59)
**Gender**		
Male	55 (93.22)	57 (96.61)
Female	4 (6.78)	2 (3.40)
**Hero type**		
Strength	11 (18.64)	10 (16.95)
Agility	28 (47.46)	41 (69.49)
Intelligence	20 (33.90)	8 (13.56)
**Lane**		
Safe	8 (13.56)	10 (16.95)
Mid	46 (77.97)	45 (76.27)
Dead	5 (8.47)	4 (6.78)
**Rank**		
None	15 (25.42)	11 (18.64)
Herald	2 (3.39)	1 (1.69)
Guardian	0 (0.00)	5 (8.47)
Crusader	9 (15.25)	4 (6.78)
Archon	12 (20.34)	8 (13.56)
Legend	8 (13.56)	12 (20.34)
Ancient	6 (10.17)	12 (20.34)
Divine	3 (5.08)	5 (8.47)
Immortal	4 (6.78)	1 (1.69)
**Total**	59 (100.00)	59 (100.00)

*N* = 118 participants.

### Confounding factors filtering

Results obtained from the independent sample t-test and crosstabs analysis showed that factors including the historical game duration [*t*(116) = −1.125, *p* = 0.263], the lane [*χ*^2^ (2) = 0.344, *p* = 0.842], and the rank [*χ*^2^ (8) = 13.772, *p* = 0.088] were all independent from the self-regulation level. Only the hero type was correlated with the self-regulation level (*χ*^2^ = 7.640, *p* = 0.022). Thus in the ensuing analysis section, we would discuss how do the two factors, i.e., self-regulation and hero type, work jointly influencing the behaviors of the players.

### Different hero types at different self-regulation levels

To explore the influence of self-regulation on the gaming behaviors of DOTA 2 players choosing different hero types, this study conducted a 2 (self-regulation: low, high) × 3 (hero type: strength, agility, intelligence) two-way ANOVA on the data of 18 behavior indicators collected during the game (see Table 1). Results showed that there were significant interactions of self-regulation and hero type on six behavior indicators, and significant main effect of self-regulation on one behavior indicator (see Table 4). These seven indicators and their definitions are as follows:

1. Number of kills: the times of the player kills opposing heroes.
2. Average number of kills: the average times of the player kills opposing heroes per minute.
3. Number of multi-kills: the times of the player has killed two or more opposing heroes within the specified time limit (within 12 s of killing the last opposing hero)
4. Proportion of the number of skills used for oneself: the ratio of the number of times the player uses skills for his/her hero to the number of times the player uses skills for all heroes.
5. Number of purchased comprehensive items: the total number of comprehensive items purchased by the player through gold.
6. Total number of team fights on the middle stage: the total number of times the player has participated in a team fight within 10–30 min after the game starts.
7. Proportion of the number of skills used for teammates: the ratio of the number of times the player uses skills for the hero controlled by his or her teammates to the number of times the player uses skills for all heroes.

**Table 4:** Results of two-way ANOVA on gaming behavior indicators.

**Behavior indicator**	**Low-level self-regulation (n = 59)**			**High-level self-regulation (n = 59)**			**Self-regulation**	**Hero type**	**Self-regulation × hero type**
	M ± SD			M ± SD			*F* (1,112)	*F* (2,112)	*F* (2,112)
	Strength	Agility	Intelligence	Strength	Agility	Intelligence			
Task performance									
Number of kills	28.09 ± 13.84	28.93 ± 9.67	40.85 ± 11.93	25.10 ± 15.00	33.00 ± 14.59	25.75 ± 10.15	2.808	1.557	4.818^**^
Average number of kills	0.83 ± 0.41	0.85 ± 0.38	1.28 ± 0.47	0.77 ± 0.50	1.06 ± 0.50	0.73 ± 0.34	1.696	1.258	5.962^**^
Number of multi-kills	11.82 ± 7.80	12.00 ± 6.43	17.50 ± 9.10	10.80 ± 8.84	15.29 ± 9.35	8.00 ± 5.15	1.785	0.649	5.103^**^
Job dedication									
Proportion of the number of skills used for oneself	0.02 ± 0.06	0.00 ± 0.00	0.04 ± 0.12	0.08 ± 0.25	0.00 ± 0.01	0.22 ± 0.26	11.264^**^	11.003^***^	5.615^**^
Number of purchased comprehensive items	6.82 ± 2.56	10.39 ± 3.33	9.05 ± 3.27	8.80 ± 4.13	7.93 ± 3.17	9.13 ± 3.27	0.037	1.414	4.151^*^
Backing up behavior									
Total number of team fights on the middle stage	9.64 ± 2.62	9.39 ± 2.27	10.10 ± 3.01	10.30 ± 1.64	10.54 ± 2.47	8.25 ± 2.25	0.001	0.929	3.155^*^
Proportion of the number of skills used for teammates	0.00 ± 0.01	0.00 ± 0.00	0.00 ± 0.01	0.02 ± 0.06	0.00 ± 0.00	0.02 ± 0.05	7.186^**^	3.386^*^	2.718

* *p* < 0.05;

** *p* < 0.01;

*** *p* < 0.001.

These seven behavior indicators are affiliated to the three categories of teamwork behaviors based on the aforementioned classifications (as in Table 1): behavior indicators (1)–(3) belong to the task performance, (4) and (5) to the job dedication, and (6) and (7) to the backing up behavior.

However, as for proportion of the number of skills used for enemy, number of destroyed defensive towers of enemy, number of times to use gold to respawn, number of purchased active items, number of consumables, total number of team fights on the initial stage, total number of team fights on the later stage, total number of placed observer wards, average number of placed observer wards, total number of placed sentry wards, and average number of sentry wards, neither the interactions of self-regulation and hero type nor the main effects of self-regulation were significant (*p*s > 0.05).

#### Task performance

##### Number of kills

**Figure 1:**
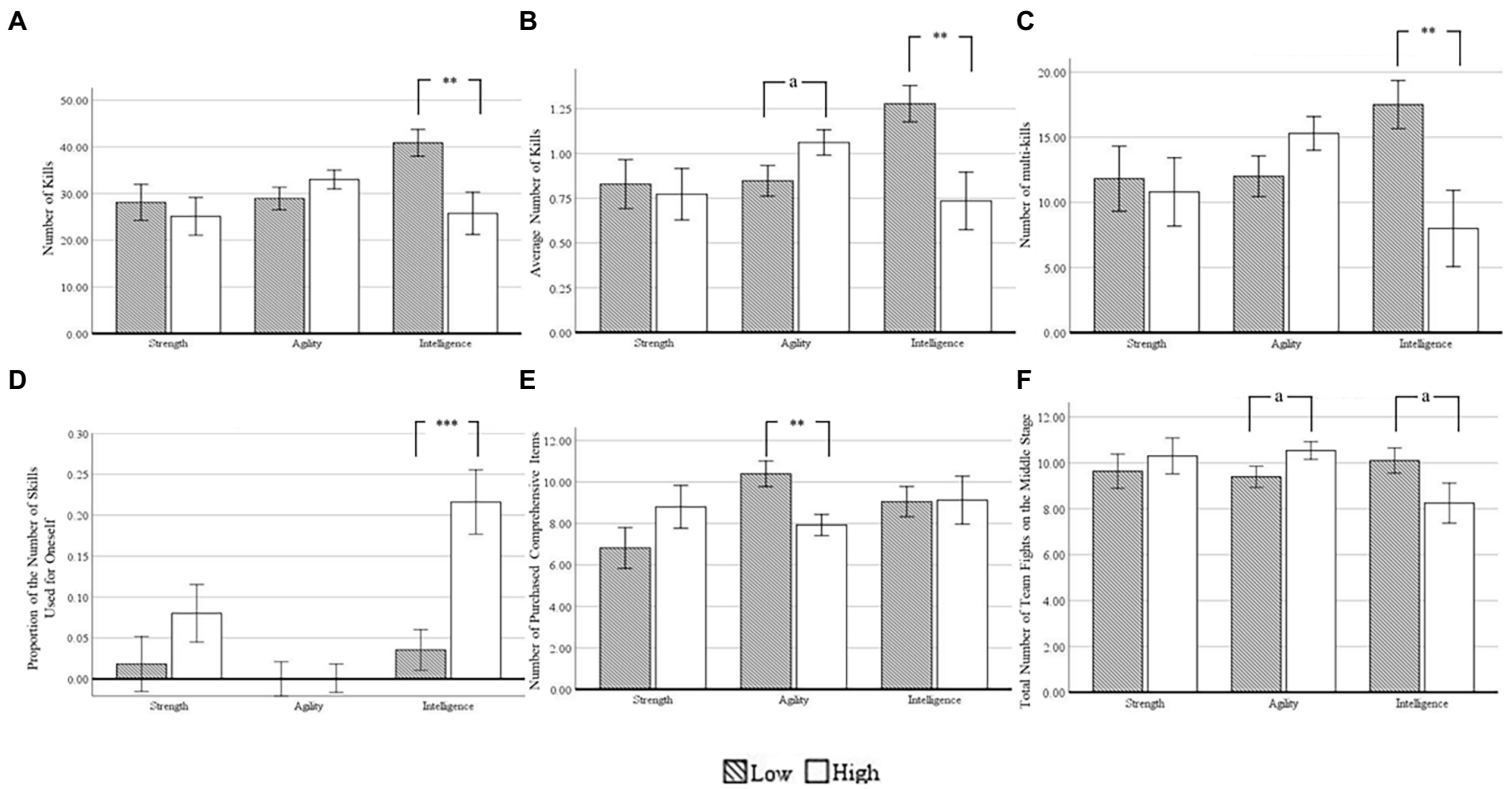
Differences between low- and high-level self-regulation group of different hero types on **(A)** number of kills, **(B)** average number of kills, **(C)** number of multi-kills, **(D)** proportion of the number of skills used for oneself, **(E)** number of purchased comprehensive items, and **(F)** total number of team fights on the middle stage. Low: the low-level self-regulation group; High: the high-level self-regulation group. The error bar represents standard error. ^a^*p* < 0.10; *p < 0.05; **p < 0.01; ***p < 0.001.

The main effect of self-regulation was not significant [*F*(1, 112) = 2.808, *p* = 0.097, η2 *p* = 0.024]. The main effect of hero type was not significant [*F*(2, 112) = 1.557，*p* = 0.215，η2 *p* = 0.027].The interaction of self-regulation and hero type was significant [*F*(2, 112) = 4.818, *p* = 0.010, η2 *p* = 0.079]. Results of simple effect analysis showed the effect of self-regulation was significant on intelligence-type heroes [*F*(1, 112) = 7.931, *p* = 0.006, η2 *p* = 0.066], but not for strength- and agility-type heroes. For intelligence-type heroes, pairwise comparison showed that players with low-level self-regulation had a significantly higher number of kills than those with high-level self-regulation (see Figure 1).

##### Average number of kills

The main effect of self-regulation was not significant [*F*(2, 112) = 1.696, *p* = 0.195, η2 *p* = 0.015]. The main effect of hero type was not significant [*F*(2, 112) = 1.258, *p* = 0.288, η2 *p* = 0.022].The interaction of self-regulation and hero type was significant [*F*(2, 112) = 5.962, *p* = 0.003, η2 *p* = 0.096]. Results of simple effect analysis showed the effect of self-regulation was not significant on strength-type heroes, marginally significant on agility-type heroes [*F*(1, 112) = 3.679, *p* = 0.058, η2 *p* = 0.032], and significant on intelligence-type heroes [*F*(1, 112) = 8.167, *p* = 0.005, η2 *p* = 0.068]. For agility-type heroes, pairwise comparison showed that players with low-level self-regulation had, with marginal significance, a lower average number of kills than those with high-level self-regulation. For intelligence-type heroes, pairwise comparison showed that players with low-level self-regulation had a significantly higher average number of kills than those with high-level self-regulation (see Figure 1).

##### Number of multi-kills

The main effect of self-regulation was not significant [*F*(1, 112) = 1.785, *p* = 0.184, η2 *p* = 0.016]. The main effect of hero type was not significant [*F*(2, 112) = 0.649, *p* = 0.525, η2 *p* = 0.011]. The interaction of self-regulation and hero type was significant [*F*(2, 112) = 5.103, *p* = 0.008, η2 *p* = 0.084]. Results of simple effect analysis showed the effect of self-regulation was not significant on strength- and agility-type heroes, but significant on intelligence-type heroes [*F*(1, 112) = 7.512, *p* = 0.007, η2 *p* = 0.063]. For intelligence-type heroes, pairwise comparison showed that players with low-level self-regulation had a significantly higher number of multi-kills than those with high-level self-regulation (*p* = 0.007; see Figure 1).

#### Job dedication

##### Proportion of the number of skills used for oneself

The main effect of self-regulation was significant [*F*(1, 112) = 11.264, *p* = 0.001, η2 *p* = 0.091]. The main effect of hero type was significant [*F*(2, 112) = 11.003, *p* = 0.00004, η2 *p* = 0.164]. The interaction of self-regulation and hero type was significant [*F*(2, 112) = 5.615, *p* = 0.005, η2 p = 0.091].Results of simple effect analysis showed the effect of self-regulation was not significant on strength- and agility-type heroes, but significant on intelligence-type heroes [*F*(1, 112) = 15.120, *p* = 0.0002, η2 *p* = 0.119]. For intelligence-type heroes, pairwise comparison showed that players with low-level self-regulation had a significantly lower proportion of the number of skills used for oneself than those with high-level self-regulation (see Figure 1).

##### Number of purchased comprehensive items

The main effect of self-regulation was not significant [*F*(1, 112) = 0.037, *p* = 0.848, η2 *p* = 0.0003]. The main effect of hero type was not significant [*F*(2, 112) = 1.414, *p* = 0.247, η2 *p* = 0.025]. The interaction of self-regulation and hero type was significant [*F*(2, 112) = 4.151, *p* = 0.018, η2 *p* = 0.069]. Results of simple effect analysis showed the effect of self-regulation was not significant on strength- and intelligence-type heroes, but significant on agility-type heroes [*F*(1, 112) = 9.471, *p* = 0.003, η2 *p* = 0.078]. For agility-type heroes, pairwise comparison showed that players with low-level self-regulation had a significantly higher number of purchased comprehensive items than those with high-level self-regulation (see Figure 1).

#### Backing up behavior

##### Total number of team fights on the middle stage

The main effect of self-regulation was not significant [*F*(1, 112) = 0.001, *p* = 0.979, η2 *p* = 0.000006]. The main effect of hero type was not significant [*F*(2, 112) = 0.929, *p* = 0.398, η2 *p* = 0.016]. The interaction of self-regulation and hero type was significant [*F*(2, 112) = 3.155, *p* = 0.046, η2 *p* = 0.053]. Results of simple effect analysis showed the effect of self-regulation was not significant on strength-type heroes, marginally significant on agility-type heroes [*F*(1, 112) = 3.570, *p* = 0.061, η2 *p* = 0.031] and intelligence-type heroes [*F*(2, 112) = 3.208, *p* = 0.076, η2 *p* = 0.028]. For agility-type heroes, pairwise comparison showed that players with low-level self-regulation had, with marginal significance, a lower total number of team fights on the middle stage than those with high-level self-regulation. For intelligence-type heroes, pairwise comparison showed that players with low-level self-regulation had, with marginal significance, a higher total number of team fights on the middle stage than those with high-level self-regulation (see Figure 1).

##### Proportion of the number of skills used for teammates

The main effect of self-regulation was significant [*F*(1, 112) = 7.186, *p* = 0.008, η2 *p* = 0.060]. The main effect of hero type was significant [*F*(2, 112) = 3.386, *p* = 0.037, η2 *p* = 0.057]. The interaction of self-regulation and hero type was not significant [*F*(2, 112) = 2.718, *p* = 0.070, η2 p = 0.046].

In terms of the other behavior indicators, i.e., the proportion of the number of skills used for enemy, the number of destroyed defensive towers of enemy, the number of times to use gold to respawn, the number of purchased active items, the number of consumables, the total number of team fights on the initial stage, the total number of team fights on the later stage, the total number of placed observer wards, the average number of placed observer wards, the total number of placed sentry wards, the average number of placed sentry wards, both main effect of self-regulation and interaction of self-regulation and hero type were insignificant (all *p* values > 0.05).

## Discussion

By analyzing real-time behavior data of players in a DOTA 2 game and controlling confounding factors that may impact gaming behaviors, we revealed how self-regulation played its part on detailed gaming behaviors in real-life teamwork settings. To study the impacts of self-regulation on players who chose different hero types, we ran a two-way ANOVA, from which we found significant differences in specific gaming behaviors of players at different self-regulation levels. These behaviors fell into various teamwork categories, including task performance, job dedication, and backing up behavior, but not including monitoring.

### Teamwork behaviors of different hero types influenced by different self-regulation levels

#### Task performance

Number of kills, average number of kills and number of multi-kills, as the typical gaming behavior indicators of task performance, can directly determine the game result in DOTA 2, and showed how well individuals can fulfill their specific responsibilities. Task performance reflects, to a certain degree, whether self-regulation successfully plays its part (Bandura and Simon, 1977; Liu, 2016). As shown in our results, when selecting agility-type heroes, players with high-level self-regulation gained higher average number of kills, showing that they delivered more damage. For players selecting intelligence-type heroes, the higher a player’s self-regulation level was, the lower number of kills, average number of kills and number of multi-kills the player would score, indicating that the players’ focus was not on killing opposing heroes. The reason behind such contrary results lay in the fact that depending on their advantages, different heroes have different primary responsibilities in DOTA 2. The primary responsibility of intelligence-type heroes is to support teammates whereas that of agility-type heroes is to deliver damage. A player’s self-regulation level may determine whether he or she can fully understand the different primary responsibility of his or her hero, thereby influencing whether he or she is able to well support the team to triumph the game. In general, the higher a player’s self-regulation is, the better the player can grasp the primary responsibility of its hero and can take more actions falling within that responsibility. Table 5 shows as players’ self-regulation increased, how their heroes scored in task performance-related behavior indicators.

**Table 5:** Changes in task performance-related behavior indicators as players’ self-regulation increased.

	**Strength**	**Agility**	**Intelligence**
Number of kills	—	—	↓
Average number of kills	—	↑	↓
Number of multi-kills	—	—	↓

— means there was no significant difference between low-level and high-level self-regulation group; ↓ means as the self-regulation increased, the behavior indicator decreased significantly; ↑ means as the self-regulation increased, the behavior indicator increased significantly.

#### Job dedication

Behaviors of job dedication reflect to what extent individuals are willing to work to fill in the gap between the status quo and the desired state (Van Scotter and Motowidlo, 1996). Though task performance-related behaviors, such as number of kills, can be impacted by a player’s willingness for job dedication, some other behaviors going beyond task performance can more purely reflect a player’s job dedication. For example, proportion of the number of skills used for oneself and the number of purchased comprehensive items were not conducted directly for completing tasks, but they showed players were making active efforts to reach the desired state. As in our results, when selecting intelligence-type heroes, players with high-level self-regulation used more skills to their own. All the skills intelligence-type heroes used for themselves were to increase their ability to better support their teammates, as the intelligence-type roles set. This showed that the higher a player’s self-regulation level is, the more dedication the player would make. Players selecting agility-type heroes tended to buy certain equipment that can enhance physical damage on the opponents, and the stronger such equipment’s capacity is, the more gold and time they cost. Frequent equipment changing usually means the poorer capacity of the equipment bought. Agility-type hero players with high-level self-regulation tended to spend longer time to accumulate more gold, and spend gold on more powerful equipment rather than easily bought cheap equipment. Such behavior also reflected these players’ better activeness to achieve the desired state. In general, players with high-level self-regulation are more willing and active to improve the status quo and the efforts they make can yield more effective results. Table 6 shows as players’ self-regulation increased, how their heroes scored in job dedication-related behavior indicators.

**Table 6:** Changes in job dedication-related behavior indicators as players’ self-regulation increased.

	**Strength**	**Agility**	**Intelligence**
Proportion of the number of skills used for oneself	—	—	↑
Number of purchased comprehensive items	—	↓	—

#### Backing up behavior

Gaming acts falling into backing up behavior, such as joining team fights and using skills for teammates, mainly reflect to what extent players rely on and support each other. The self-regulation ability can help individuals analyze their tasks, understand responsibilities of their own and their teammates, help teammates realize their goals while working towards their own, so as to get closer to the shared goal (Gladstein, 1984; Miller and Brown, 1991; Campion et al., 1993; Neal and Carey, 2005). As in our results, players with high-level self-regulation better understood teammates’ needs and used more skills for their teammates to give them timely support. In addition to using more skills for teammates, players with high-level self-regulation also tended to better cooperate with their teammates while team fights happened. We found that agility-type heroes, as the core of the team fight, more frequently participated in team fights to deliver physical damage on the middle stage when their teammates needed to accumulate experience and gold; meanwhile, intelligence-type heroes usually supported individual heroes to fight rather than directly participating in team fights on the middle stage. In short, players with high-level self-regulation seem to better grasp the responsibilities and status quo of their teammates and their own, and take better supportive actions. Table 7 shows as players’ self-regulation increased, how their heroes scored in backing up behavior-related behavior indicators.

**Table 7:** Changes in backing up behavior-related behavior indicators as players’ self-regulation increased.

	**Strength**	**Agility**	**Intelligence**
Total number of team fights on the middle stage	—	↑	↓
Proportion of the number of skills used for teammates	↑	↑	↑

As shown in the results above, self-regulation had totally different impacts on the gaming behaviors, when players selected different types of heroes. It seems that in real-life teamwork such as DOTA 2, self-regulation enables players to play their parts well, so that they can fulfil more responsibility-oriented tasks (representing task performance), be more active in making effective efforts to improve themselves (representing job dedication), and provide more timely support to teammates (representing backing up behavior).

In the present study, we did not find significant behavioral difference between players with high and low self-regulation level in monitoring, such as placing observer wards and sentry wards. One possible reason is that self-regulation does not have direct impact on monitoring behaviors, and the promoting effect of self-regulation on teamwork performance is realized through the influence of task performance, job dedication and backing up behavior. Another possible reason is the complexity of monitoring the behavior itself. In a team, monitoring means that an individual is highly sensitive to clues in a cooperative environment, and adjusts his or her behaviors in time (Lennox and Wolfe, 1984). For the teamwork behaviors, monitoring contains more implicit components than other categories, which is more difficult to use observable indicators such as performance to evaluate (Gangestad and Snyder, 2000). Since the explicit behaviors, such as placing wards, reflect the individual’s attention to the external environment mostly, it is difficult to show the internalization process of monitoring. Due to such complexities, impacts of self-regulation on monitoring may not be seen in typical explicit behaviors of monitoring in DOTA2—placing observer wards and sentry wards.

Our findings showed the specific impacts of self-regulation on detailed gaming behaviors, and probably explains why stronger self-regulation can bring about better teamwork results as shown in previous researches (Sachdeva et al., 2009). In terms of task performance-related behaviors, self-regulation impacted how players used kills to destroy the enemy with efficiency; in terms of job dedication-related behaviors, self-regulation could decide the willingness of players to make active efforts; as for backing up behavior, self-regulation could determine how frequent players take interpersonal facilitation actions to support teammates in need. However, we did not see the impacts of self-regulation on monitoring behaviors in our research, probably because self-regulation has no direct impacts on such behaviors, or because monitoring behaviors are too complex. From the results, we can conclude that players with stronger self-regulation ability can better understand the responsibilities of their own and their teammates and can more accurately analyze tasks, so as to take more targeted actions to help achieve team goals.

### The practical implications of self-regulation assessment

This study demonstrated that self-regulation, as a psychological characteristic, presented a consistent behavioral concept in the actual teamwork behavior, and this behavioral concept would produce specific behavioral differences according to the role’s different responsibilities. There could be some differences in concrete behaviors from different settings, nevertheless, considering that self-regulation plays an indispensable role in teamwork (Priest et al., 2002), and similar categories of teamwork behaviors exist in all these real-life settings (Cleveland et al., 1989), our findings in DOTA 2 could be extended to other settings as well. Take for instance the category of task performance, the number of kills in DOTA 2 is approximately similar to the number of productions in organization (Borman and Motowidlo, 1993). Moreover, the division of responsibilities is everywhere, whether in real life or in the virtual world, for example, the hero type is similar to the department of company. Such finding that self-regulation had great impacts on teamwork performance in hero world of online gaming could also be transferred to teamwork in face-to-face settings. Usually in daily life, we would like to select people with higher self-regulation or improve team members’ self-regulation ability to promote better teamwork (Tschan, 1995, 2002). Our findings on the impact of self-regulation on concrete behaviors may shed some light on how we can better give play to self-regulation in offline teamwork scenarios:

1. As we can see in our research, self-regulation had different impacts on different categories of teamwork behavior. Behaviors of some teamwork categories were more likely to be impacted by one’s self-regulation ability than others. Given that, we may need to distinguish different categories of teamwork behavior or different type of task, when selecting team members considering their self-regulation.
2. Our research also found that the impacts of self-regulation on teamwork were also related to what roles individuals played in the team. Players with greater self-regulation ability adopted behavior patterns that were more in line with their roles, suggesting that self-regulation may bring some general advantages in real-life teamwork. On the other hand, for certain heroes such as strength-type heroes, few differences can be seen in the players’ gaming behaviors with different self-regulation levels. This may indicate that when selecting candidates for certain team roles offline, we do not necessarily have to consider their self-regulation ability.

By studying how players selecting different hero types with different self-regulation levels behave in DOTA 2, we revealed in detail how self-regulation can influence different categories of teamwork behaviors according to different responsibilities in a real-life case, making a further step from previous researches that rarely used real-life teamwork cases and lacked detailed behavioral observation. DOTA 2 is a game world teeming with teamwork, where players need to face and adapt to many changes and conflicts with their teammates, and cooperate with each other to achieve team goals (Rigby and Przybylski, 2009). Such real-life teamwork cases can truly present the impacts of psychological factors without being disturbed by any man-made settings (Lv and Su, 2005); moreover, data extracted from real-time recordings are more objective than those obtained through the self-reporting approach (Veenman et al., 2004; Veenman, 2011). In short, by comparing and analyzing the behaviors of various DOTA 2 heroes selected by players with different self-regulation levels, we further understand how psychological factors such as self-regulation impacts different categories of teamwork. Our findings could be applied to face-to-face teamwork scenarios, where different hero types correspond to the differences of the professional division of work, including different occupations, identities and responsibilities. Our findings implicates that in real life, self-regulation enables individuals with different identities and responsibilities to clearly set their own goals fitting current status of the team, and to constantly adjust their teamwork behaviors in certain ways to approach the goal. Our research also paves a new path for conducting fine-grained studies about individual behaviors and psychological mechanisms in real-life teamwork cases, hopefully improving our interpretation of the behaviors and mentality of team members in complex teamwork.

### Limitations and future research

This research revealed the impacts of self-regulation on specific real-life teamwork behaviors using detailed DOTA 2 behavior data, but it still has some limitations. First, DOTA 2 users are mainly young males as shown in an official DOTA 2 report (Into the Breach Esports, 2021). This means that the research results from the DOTA 2 teamwork case may not be well applied to other scenarios with different age and gender structures. Second, this paper only looked at typical teamwork-related monitoring behaviors in DOTA 2, and did not find clear differences in these behaviors on players with different self-regulation levels. Subsequent researches may need to expand behavior indicators, which may help determine whether self-regulation can impact monitoring behaviors in teamwork. Third, this paper only made a broad classification of responsibilities of different types of heroes. In future research, we can look into such responsibilities in detail, so as to further explore the role of self-regulation in game teamwork scenarios. In addition, future research could align MOBA game roles with offline roles, in a bid to better apply the research results in everyday life.

## Conclusion

By analyzing teamwork behavior data of players in a DOTA 2 game, this paper explored how self-regulation impacted various specific teamwork behaviors of different categories, when players selected different heroes. As shown in our results, the impacts of self-regulation can be seen in various teamwork behavior categories including task performance, job dedication and backing up behavior, but not including monitoring behaviors. Moreover, self-regulation had different impacts on the behaviors of different heroes as different types of heroes bear different responsibilities and have developed respective behavioral patterns. In short, self-regulation can help individuals better understand the responsibilities of their own and their teammates and take actions in line with their responsibilities, so as to more effectively help the team achieve shared goals. By revealing the impacts of self-regulation on concrete gaming behaviors to improve teamwork performance, this paper shed some light on how we can give play to self-regulation in real-life teamwork scenarios.

## Data availability statement

The original contributions presented in the study are included in the article/supplementary material, further inquiries can be directed to the corresponding author: NZ, zhaonan@psych.ac.cn.

## Ethics statement

The studies involving human participants were reviewed and approved by Scientific Research Ethics Committee of Institute of Psychology, Chinese Academy of Sciences (ethical code H15010). The patients/participants provided their written informed consent to participate in this study.

## Author contributions

NZ contributed to the conception, design of the study, and to the final version of the manuscript. YZ, WC, and FX were responsible for data collection. JL gave the detailed explanation of gaming behavior indicators and drafted the discussion part of the manuscript. YW performed the statistical analysis and wrote the manuscript with input from all authors. All authors contributed to the article and approved the submitted version.

## Funding

This work was financially supported by the Strategic Priority Research Program of Chinese Academy of Sciences (No. XDC02060300) and Youth Innovation Promotion Association CAS.

## Conflict of interest

The authors declare that the research was conducted in the absence of any commercial or financial relationships that could be construed as a potential conflict of interest.

## Publisher’s note

All claims expressed in this article are solely those of the authors and do not necessarily represent those of their affiliated organizations, or those of the publisher, the editors and the reviewers. Any product that may be evaluated in this article, or claim that may be made by its manufacturer, is not guaranteed or endorsed by the publisher.

## Footnotes

^1^ https://www.opendota.com
